# Percutaneous thrombolysis via cholecystostomy catheter to dissolve biliary clots causing obstructive jaundice

**DOI:** 10.1093/jscr/rjae055

**Published:** 2024-02-22

**Authors:** Sonya A Smith, Fraser Simpson, Nicholas Bell-Allen, Nicholas Brown, Sanjivan Mudaliar, Khurram Aftab, Diana Tam, Manju D Chandrasegaram

**Affiliations:** Department of General Surgery, The Prince Charles Hospital, 627 Rode Rd, Chermside QLD0, Queensland 4032, Australia; Department of General Surgery, The Prince Charles Hospital, 627 Rode Rd, Chermside QLD0, Queensland 4032, Australia; Department of General Surgery, The Prince Charles Hospital, 627 Rode Rd, Chermside QLD0, Queensland 4032, Australia; Department of Radiology, The Prince Charles Hospital, 627 Rode Rd, Chermside, Queensland 4032, Australia; Department of Gastroentrology, The Prince Charles Hospital, 627 Rode Rd, Chermside, Queensland 4032, Australia; Department of Radiology, The Prince Charles Hospital, 627 Rode Rd, Chermside, Queensland 4032, Australia; Department of General Surgery, The Prince Charles Hospital, 627 Rode Rd, Chermside QLD0, Queensland 4032, Australia; Department of General Surgery, The Prince Charles Hospital, 627 Rode Rd, Chermside QLD0, Queensland 4032, Australia

**Keywords:** haemobilia, thrombolysis, percutaneous cholecystostomy

## Abstract

Haemobilia, or bleeding within the biliary tree, is rare. It can cause biliary obstruction secondary to blood clots. A comorbid 87-year-old was admitted to hospital with acute cholecystitis, choledocholithiasis, and an *Escherichia coli* bacteremia. He had a partial pancreatectomy and gastrojejunostomy 35 years prior for severe pancreatitis. He was treated with antibiotics and a percutaneous cholecystostomy. He developed atrial fibrillation and was subsequently commenced on warfarin. He re-presented 5 days after discharge with abdominal pain and fevers. Liver function tests revealed cholestasis and a supratherapeutic international normalised ratio. Imaging showed cholecystitis, biliary obstruction, and extensive biliary blood clots. He improved with antibiotics, vitamin K, and alteplase flushes through the percutaneous cholecystostomy. Repeat cholangiogram demonstrated dissolution of the biliary clots. Due to altered anatomy and comorbidities, alteplase flushes were utilized to relieve this patient’s biliary obstruction. Thrombolytics may assist in treating biliary clots when first-line options are not possible or favourable.

## Introduction

Haemobilia, or bleeding into the biliary tree, is rare. Haemobilia is usually iatrogenic and secondary to percutaneous procedures involving the liver [1]. Less common causes include trauma, malignancy, gallstones, and coagulopathy [1]. Approximately half is extrahepatic, arising from the common bile duct (CBD) or gallbladder [2]. Bleeding is usually minor, but cases of major hemorrhage have been reported [2].

Bile inhibits the formation of a fibrin clot. However, when haemobilia occurs slowly, blood separates from bile to form clots; rarely, these clots can cause biliary obstruction [3]. Haemobilia characteristically presents with a triad of melena, jaundice, and right upper quadrant (RUQ) pain [2]. Uncommonly, it presents as cholangitis or cholecystitis secondary to biliary obstruction [2].

The management of haemobilia depends on etiology and sequelae. Coagulopathy should be corrected, and biliary obstruction should be relieved. Haemobilia is often managed conservatively. In one study, 43% of 171 cases were managed conservatively [2]. If bleeding is from the gallbladder or if haemobilia is complicated by cholecystitis, urgent cholecystectomy is recommended [2]. Biliary clots are often removed by endoscopic retrograde cholangiopancreatography (ERCP); however, endoscopic procedures can be difficult and lengthy if anatomy is altered, such as in patients with a gastrojejunostomy [4].

Thrombolytics maintain patency in venous access devices and break down clots to restore blood flow, such as in acute myocardial infarctions [5–7]. The use of thrombolytics to break down biliary blood clots has been rarely described in the literature. In a post-operative liver transplant patient, alteplase was utilized to successfully clear obstructing biliary blood clots, after ERCP was unsuccessful [8]. Alteplase was flushed through the biliary tree via a nasobiliary catheter, relieving the obstruction [8]. Another case describes acute cholangitis secondary to biliary blood clots in the context of haemobilia from a percutaneous cholecystostomy; saline was flushed through the cholecystostomy to successfully relieve the obstruction [9].

## Case report

An 87-year-old presented with 3 days of RUQ pain, fevers, and nausea. Comorbidities included chronic obstructive pulmonary disease, pulmonary hypertension, ischaemic heart disease, mitral stenosis, chronic kidney disease, and gastroesophageal reflux. His surgical history included a partial pancreatectomy, partial gastrectomy and gastrojejunostomy, secondary to alcoholic pancreatitis complicated by a pancreatic abscess over 35 years ago.

**Figure 1:**
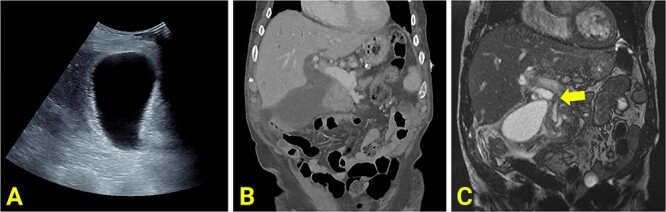
(A) Ultrasound scan showing a distended and enlarged gallbladder with a thickened wall suggestive of acute cholecystitis. No obvious gallstones are present. (B) Reconstructed contrast-enhanced coronal CT shows an enlarged and distended gallbladder, a thickened gallbladder wall, pericholecystic fluid, and a dilated common hepatic duct. (C) MRCP showing an obstructed gallstone at the confluence of the common hepatic duct, common bile duct and cystic duct (marked with arrow).

Bloods on admission revealed the following: haemoglobin (Hb) 141 (130–180 g/L), white cell count (WCC) 23.7 (4–11 × 10^9^/L), bilirubin total/direct 35/25 (<20/<7 μmol/L), alkaline phosphatase (ALP) 383 (30–110 U/L), gamma-glutamyl transferase (GGT) 267 (<55 U/L), alanine transaminase (ALT) 738 (<35 U/L), and aspartate aminotransferase (AST): 884 (<40 U/L). Initial imaging suggested acute cholecystitis; ultrasound (US) showed a distended gallbladder with a thickened wall (Fig. 1A), and non-contrast computed tomography (CT) showed a distended gallbladder with pericholecystic stranding (Fig. 1B). One day later, magnetic resonance cholangiopancreatography (MRCP) showed cholecystitis, biliary duct dilatation, and choledocholithiasis (Fig. 1C). No cholelithiasis was present. Blood cultures were positive for *Escherichia coli*.

**Figure 2:**
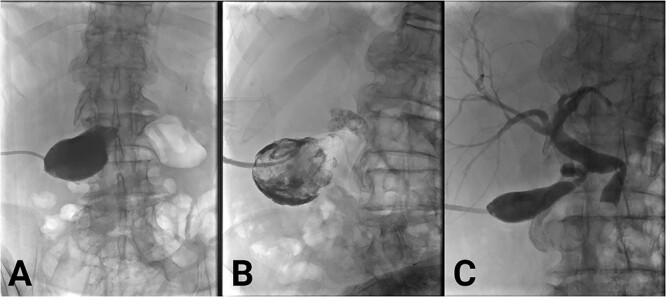
(A) Initial cholecystogram showing contrast filling the gallbladder. (B) Subsequent cholecystogram showing diffuse filling defects consistent with clot in the gallbladder and cystic duct. (C) Final cholecystogram after the use of alteplase showing resolution of clots and contrast flowing into the biliary tree.

He received intravenous antibiotics, and, due to multiple comorbidities, was managed non-operatively with a percutaneous cholecystectomy. Positioning of the cholecystectomy tubing was confirmed with a cholangiogram (Fig. 2A). He developed valvular atrial fibrillation and commenced on warfarin. The sepsis resolved. He was discharged after 9 days.

**Figure 3:**
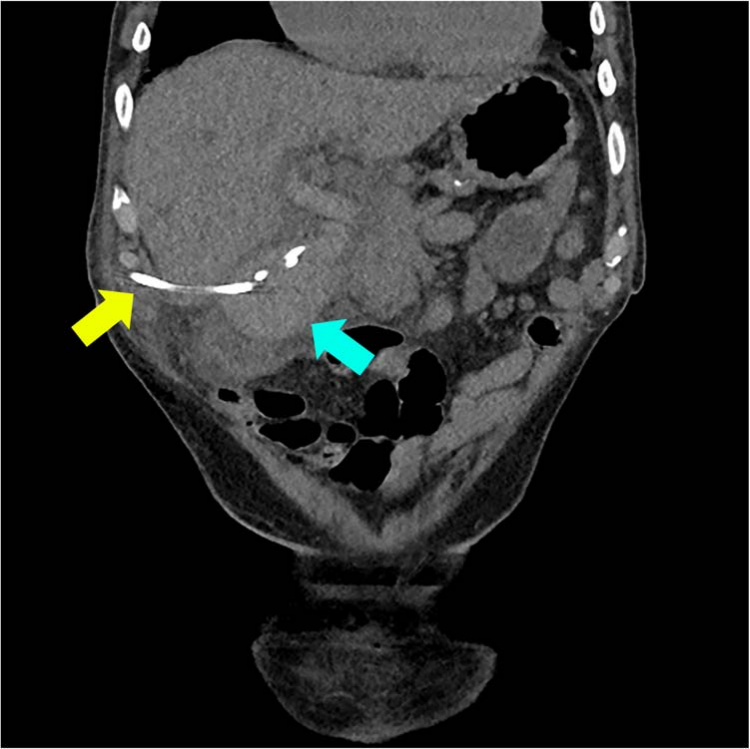
Coronal CT reconstruction showing high density material within the lumen of the gallbladder suggestive of haemorrhage (marked with blue arrow) and cholecystostomy tube (marked with yellow arrow).

He represented 5 days later with blood draining from the percutaneous cholecystostomy, RUQ pain, and fevers. Two days prior, in the community, international normalised ratio (INR) was 6.2. Bloods on admission included: Hb 118, WCC 23.5, INR 2.4, bilirubin 24/18, ALP 457, GGT 441, ALT 179, and AST 328. CT showed a distended gallbladder with pericholecystic fat stranding and intra- and extrahepatic duct filling defects suggestive of haemobilia (Fig. 3).

Warfarin was withheld. He was given Vitamin K and antibiotics. Over the next day, there was minimal cholecystostomy output. Pain was ongoing. On Day 2 of admission, bloods were as follows: Hb 113, WCC 20.2, INR 4.2, Bilirubin (total/direct) 61/50, ALP 730, GGT 456, ALT 412, and AST 520. A cholecystogram (Fig. 2B) Day 3 of admission confirmed ongoing biliary obstruction.

Management options were discussed with the patient, including risks and benefits of alteplase flushes. The patient consented to alteplase flushes. For 5 days, he received daily flushes, 1 mg alteplase diluted in 5 ml of saline, through the cholecystostomy tubing, which was clamped for 10 minutes afterwards.

Over 5 days, his pain resolved and liver function improved. The drain began to empty, initially blood-stained, followed by clear bile. Cholangiogram demonstrated dissolution of the intrabiliary clots (Fig. 2C). The choledocholithiasis remained. On discharge, the percutaneous cholecystectomy was draining bile freely. Warfarin was withheld indefinitely after cardiology input. After two unsuccessful endoscopic attempts to remove the choledocholithiasis with balloon ERCP, it was removed successfully by interventional radiology-guided lithotripsy. Due to comorbidities and absence of gallstones on repeat cholangiogram, the patient did not proceed to cholecystectomy. He was given ursodeoxycholic acid to prevent gallstone formation. Cholecystostomy tube was removed. He remains independent at home post lithotripsy.

## Discussion

This patient’s haemobilia was likely multifactorial. The bleeding may have occurred from the gallbladder at the area of the percutaneous cholecystostomy. It may have also occurred due to irritation of the CBD from choledocholithiasis. It is likely that a supratherapeutic INR secondary to warfarin potentiated the bleeding, compounded by potential vitamin K deficiency secondary to biliary obstruction. Only a few reported cases of haemobilia due to coagulopathy exist in the literature; in one case series, only 4 of 222 cases, or 2%, were caused by a coagulopathy [2]. In the same series, nine cases originated from the gallbladder, such as haemorrhagic cholecystitis [2].

Haemobilia is often treated by embolization or endoscopic management. However, in our patient, no obvious source of active bleeding was found, and thus this case was not amenable to embolization. As well, our patient was not amenable to a standard ERCP given his previous surgery; endoscopic access was difficult given his altered anatomy of a long limb gastrojejunostomy. Due to altered anatomy, endoscopic procedures can be complex and prolonged, with increased anaesthetic time [4]. This patient had a high anesthetic risk due to comorbidities. He was deemed too high risk for a surgical approach which led the team to use options that were less invasive to ultimately manage his bile duct stone while managing his complex comorbidities. In summary, alteplase flushes may be a treatment option for biliary obstruction secondary to haemobilia, when first-line options are not possible or favourable.
